# Neurasthenia, or Nervous Exhaustion

**Published:** 1889-01

**Authors:** Floyd S. Crego

**Affiliations:** Buffalo; Professor of Nervous Diseases and Insanity, Medical Department, Niagara University; Senior Attending Physician to Providence Asylum for the Insane, &c.; 280 Franklin Street


### *NEURASTHENIA, OR NERVOUS EXHAUSTION.*

By FLOYD S. CREGO, M. D., Buffalo.

Professor of Nervous Diseases and Insanity, Medical Department, Niagara University; Senior Attending Physician to Providence Asylum for the Insane, &c.

It is but a short time since Beard first directed the attention of the profession to a disease which had for some time been the subject of investigation by him. He gave to it the name neurasthenia, and described the various symptoms of the malady. The chief symptoms complained of were those which were due to the exhaustion of the various parts of the nervous system. He said: "The sufferers from the disease are counted by the thousands, and, in fact, hundreds of thousands, in this country." The brain-workers of the northern and eastern portions of this country are the chief sufferers. It is not, however, limited to this class, for physical overwork is a powerful factor in its causation.

When Beard first gave his writings to the profession, on the subject, he was violently assailed and derided; but, through the efforts of Erb, in Germany, and many others, he has long since received just recognition for his study and research. When Erb read his writings, he is said to have remarked: "I know of dozens of such cases," and he has since adopted the term, as have most of the authorities on the subject. Many of the patients observed suffer chiefly from symptoms referable to the brain, and in many, again, the spinal cord seems to be the seat of the disease. Ross has divided the subject into neurasthenia cerebralis, and neurasthenia spinalis. To the first he gives a definition as follows: "Neurasthenia is a functional disease, in which numerous and well-marked, although variable, symptoms supervene, simultaneously and successively, under conditions in which the nervous system is subjected to severe strain from overwork or other cause." To neurasthenia spinalis he gives the following definition: "Neurasthenia spinalis is observed in patients who are subject to the general symptoms grouped under the popular name of nervousness, but in this affection the functions of the cord are affected in a special degree." To avoid the use of this name, and to avoid "specialization," as it is called, many physicians have assigned the grouping of symptoms to various maladies, such as hysteria, hypochondria, and many have insisted it is a form of insanity, some melancholia, and again it has been called paranoia. It is the forerunner of insanity, and many patients remain on the border-line before insanity is really established. If we are able to avert this evil, so much the better for them and for medical science.

Those who have studied nervous diseases to any great extent will at once see the folly of calling this disease hypochondria; neither can it be called hysteria. This disease is most frequent in males, while hysteria affects females chiefly. It has been described under the head of melancholia; but, while we know that sufferers from this malady have morbid fears, we know that they do not have delusions. If these fears seem at times to control them, they are easily dispersed by argument, and patients admit the folly of their notions, which is not the case with delusions due to insanity. Some writers have attempted to give several divisions of this variety of neurasthenia, founded upon the cause, but, as the symptoms and course are about the same, this seems to be unnecessary and purely arbitrary.

For the cause, in many cases, we must look, as we would naturally suppose—this being essentially an American disease—to our own habits and customs. In no country in the world is there such a great demand on any one individual, social and business, as in our own. The business man, the lawyer, and other professional men, perform their business duties in such a manner as to subject the nervous system to the greatest possible strain. They carry their labor far into the evening, and their social engagements follow so closely and persistently, that their nervous system is undermined. This is in marked contrast to the customs in other countries, where the business man does little in the so-called social line, and the man of society is never obliged to attend to business cares. This is not saying that the man of business in other countries does not take recreation and have social enjoyments. He does most decidedly, but it is of a kind not to strain the nervous apparatus beyond endurance. Add to this the entire lack of physical training and want of proper exercise, and you have the starting point of most of these cases in men. From such a parent, a child must necessarily inherit an unstable nervous apparatus. The training of the child at home, and especially at school, where little or no attention is paid to physical culture and development, but too great mental effort is forced upon them, is the second factor in the causation. The disease is said to be inherited, and in most cases is traced to the existing evils in our habits and customs. This disease is frequently inherited from parents who have graver forms of nervous trouble, such as epilepsy, megrim, insanity, etc. It is due to shock and great mental excitement. It follows childbirth, and is frequently caused by uterine disease. This group of symptoms is considered by some to be a part of certain forms of uterine disease, and the uterus is treated, and treated without avail. This fact has been especially brought to our attention by Prof. Goodell, in an article on Nerve-counterfeits of Uterine Disease; and in order that this source of error may be corrected, I quote from his article to some extent:

"The crying medical error of the day is, in my opinion, the mistaking of nerve-disease for womb-disease. From this widespread delusion, it has come to pass that no organ in the human body is so over-treated and, consequently, so maltreated, as the womb. Fine lesions of nerve-ganglia are hard to make out, however exacting their symptoms. Take, for instance, insanity or epilepsy; even in the dead-room their lesions often elude our instruments of precision. But the womb, unfortunately, being reachable, seeable, and directly treatable, is charged with almost all the ills that female flesh is heir to; and it is, too often, made the scapegoat for headaches and nape-aches, for spine-aches and backaches, and for various other so-called uterine symptoms, which may be due solely to nerve-exhaustion, or malnutrition of nerve-centers, and not to reflex action from some real or some supposed uterine disorder. Then, again, misled by traditional teaching, by such a name as woman (womb-man), by such a misnomer as hysteria (womb-disease), we yoke our practice to theory. So, whenever we find a train of *hysterical* symptoms associated with a disordered or displaced womb in a *womb-man*, we jump with doubled energy to the conclusion that the uterine lesion is not a symptom, or a sequence, or a coincidence, but the factor, and at once proceed to treat it accordingly. Then, again, forgetful that the imponderables are great forces in nature, that a single mental stimulus to unstable nerve-molecules will awaken many reflexes, we overlook the tyranny of woman's over-sensitive organization, and underrate the influence of nerve perturbations or of psychical disturbances."

Undoubtedly, too, cases of uterine disease are overlooked by the physician, and this is occasionally a source of error. The much-abused subject of lithemia has been put forward as a factor in its causation; and while the disease does occur to a degree in these persons, we must avoid too sweeping statements on this subject. Certain is it that overwork and want of physical exercise beget a condition which gives rise in time to an unstable nervous mechanism, and finally nervous exhaustion; and this, as we would suppose, is an efficient cause in most of the cases.

"All diseases, such as dyspepsia and heart disease, which diminish the nutrition of the nervous system and the body generally, act as producing causes, though it must be remembered that diminished nutrition does not itself give rise to this affection."

In the same manner, constipation acts to produce this malady.

In marked contrast to those who overwork, are those who do nothing, and this disease seems to attack this class of people who may be called idlers; women who do not assume the care of their households; do nothing but try to enjoy themselves; and those who, even though they make the effort, gradually become unable to do anything. Men also who are unwilling to do anything but amuse themselves, gradually fall into this condition. They seem to exhaust their nervous power by studying the art of doing nothing, and yet occupying their time. Alcohol is also a potent factor in the etiology.

The symptoms of the disease are so variable, that it is almost impossible to give them in any regular order. By far the most frequent symptom, and one observed early in the disease, is languor and disinclination to exertion, and great exhaustion following any extraordinary effort, either mental or physical. The head aches, or, at least, feels uncomfortable. Sometimes it is described by patients as if very full, and again the opposite. If we examine the head by pressure, we will find many tender spots. Patients will tell you that when the head does not actually ache these spots will burn. There is great pain in the back of the head and upper part of the neck. When this is severe, it is attended by motor and sensory disturbances in the extremities. They sleep poorly and are exceedingly irritable. The least noise or excitement about the house or office makes them excitable and restless. Owing to indigestion and loss of appetite, they lose rapidly in flesh. Vomiting and purging are frequent symptoms. Again, patients are obstinately constipated. Ringing noises in the ears, especially when they lie down at night, is a distressing and constant symptom. They complain of vertigo, so extreme at times that they almost fall down. Irregularity of the heart's action is common, the pulse varying in the same person, in a short time, without any apparent cause, from 120 beats per minute to fifty-four. The patient has morbid fears, and becomes so apprehensive that he is unwilling to go away from his house; has a dread of high or open places. The vaso-motor disturbances are very distressing; the patient notices at times he is exceedingly pale, and, again, excessively flushed, and it is said that they have pallor on the one side of the face and flushing on the other. The hands and feet are alternately hot and cold. They complain frequently of pain in the extremities, and a sense of great weight in them. The disturbance of vision is so great that patients seem to have hallucinations. Black specks constantly floating before the eyes is an early symptom, and one of the last to disappear. The countenance changes from cheerful to melancholic or apprehensive. The pain is in the back of the head the condition be associated with general anemia, as it usually is, and where hyperemia is present, there is a pressure at the top of the head. Alteration in the tone of the voice is noticeable. "A complaining, weak, high-pitched voice is frequently met with, owing to instability of the nervous system." The peculiar effect that changes of weather has on such patients is very noticeable. All grades of this disease will come under our care from time to time. Some will be little affected, while others will be completely prostrated by it. All demand attention. Most can be relieved by constant and persistent care and treatment.

As to treatment, our patience and skill will be taxed to the uttermost in the care of this disease, and no two patients can be treated precisely alike. The proper regulation of the diet will be the first step, for in no disease is the appetite so capricious as in this. Patients will eat sparingly at times, and when the notion takes them they will eat to excess, and usually of a quality of food most harmful to them. In this, as in most nervous troubles, patients do better on the milk diet, taking one to three quarts daily, if possible. Koumiss, peptonized beef, Matzoon and other prepared foods will be of great service. Rest in bed may be necessary, or it may be advisable to give the patient moderate exercise, gradually increasing it. It is a nice point to determine whether this form of exercise is advisable, or complete mental and physical rest after the plan of Weir Mitchell, is indicated. It undoubtedly depends on the degree of exhaustion caused by patients seeing friends and doing a certain amount of mental work, as they necessarily must, if they take much out-of-door recreation.

Where the plan of exercise is determined upon, care should be taken to avoid too great exertion at first. It should be regular, and plenty of mental and physical rest given in the intervals. When the Weir Mitchell plan is undertaken, it should be remembered that little is accomplished without following carefully every instruction given. Complete rest—mental and physical—with the patient in almost complete seclusion, massage and electricity are given daily, and, at the same time, large quantities of milk and light foods are administered several times a day. As to remedies, it will be impossible to enumerate all these we will find useful. Iron and cod-liver oil are the two which stand at the head; an elixir, of gentian, with the pyro-phosphate of iron, meets the indications well. Cod-liver oil, with Rose's peptonized beef, has proved to be of great service in nervous troubles. Maltine with cod-liver oil seems to act as well in some cases, and often phosphorus and phosphate of lime are useful. Some physicians extol the use of zinc in the form of oxide or valerianate. In many cases it has a wonderful effect, and this has so long been recognized by the profession that the various drug-houses have prepared a pill of valerianate iron, quinine, and zinc, which is very serviceable. Dr. Webber, of Boston, recommends Val. Zinci, gr. 2; Ext. Hyoscyamii, Ext. Conii, each, gr. ½, three times daily. It has the advantage of being sedative and slightly laxative. Dr. Sachs, of New York, recommends Phosphide of Zinc, gr. 1/6, Quinine Sulph., gr. 1 1/4, and Sulph. Strychnia, gr. 1-60—t.i.d. If the patient is constipated, he adds 1/4 gr. Aloin at night. To relieve constipation, Rhamnus, Enonymus, or Aloin seem to be most generally used. Camellia in the form of the fluid extract, brucia combined with digitalis in a tablet triturate, caffeine in the form of the citrate, will be found to have a place in the treatment. In females, a combination of iron and ergot is frequently indicated. "Just how ergot acts in nervous diseases is not known, nor, indeed, are we likely to know, in satisfactory detail, the action of many remedies. That ergot contracts the blood-vessels is one of the established facts and one of the few and definite foundations in therapeutics; but that this is the only effect of this drug on the body, no student of nervous troubles will claim." To give rest and to produce sleep, we find it necessary to resort to a sedative. While many are recommended, the only ones I wish to especially call your attention to is Urethan, and the Elix. Val. Ammonia with Bromide Potassium, one dram of the Elixir and ten grains of the Bromide, given every few hours, have proved a boon in this affection. The use of the hypophosphites, nux vomica and its alkaloids, galvanic and faradic electricity, is so well established, and so thoroughly understood, that they need no mention here. It is useless, too, for me to add that any affection, such as disease of the uterus, constipation, etc., which cause reflex nervous symptoms, must be relieved before we can expect a favorable result.

280 Franklin Street.

